# The Glycemic Control Potential of Some Amaranthaceae Plants, with Particular Reference to In Vivo Antidiabetic Potential of *Agathophora alopecuroides*

**DOI:** 10.3390/molecules27030973

**Published:** 2022-02-01

**Authors:** Elham Amin, Mohamed Sadek Abdel-Bakky, Mostafa Assem Darwish, Hamdoon A. Mohammed, Sridevi Chigurupati, Kamal Ahmad Qureshi, Marwa H. A. Hassan

**Affiliations:** 1 Department of Medicinal Chemistry and Pharmacognosy, College of Pharmacy, Qassim University, Buraydah 51452, Saudi Arabia; ham.mohammed@qu.edu.sa (H.A.M.); S.Chigurupati@qu.edu.sa (S.C.); 2 Department of Pharmacognosy, Faculty of Pharmacy, Beni-Suef University, Beni-Suef 62514, Egypt; marwa.hassan@pharm.bsu.edu.eg; 3 Department of Pharmacology and Toxicology, College of Pharmacy, Qassim University, Buraydah 51452, Saudi Arabia; abdelbakkym@yahoo.com; 4 Department of Pharmacology and Toxicology, Faculty of Pharmacy, Al-Azhar University, Cairo 11751, Egypt; 5 Department of Pharmacology and Toxicology, Faculty of Pharmacy, Nahda University, Beni-Suef 11787, Egypt; mostafa.darwish@nub.edu.eg; 6 Department of Pharmacognosy, Faculty of Pharmacy, Al-Azhar University, Cairo 11751, Egypt; 7 Department of Pharmaceutics, Unaizah College of Pharmacy, Qassim University, Unaizah 51911, Saudi Arabia; ka.afrah@gmail.com

**Keywords:** *Agathophora alopecuroides*, α-amylase, α-glucosidase, in vivo antidiabetic activity, STZ

## Abstract

Natural products continue to provide inspiring moieties for the treatment of various diseases. In this regard, investigation of wild plants, which have not been previously explored, is a promising strategy for reaching medicinally useful drugs. The present study aims to investigate the antidiabetic potential of nine Amaranthaceae plants: *Agathophora alopecuroides*, *Anabasis lachnantha*, *Atriplex leucoclada*, *Cornulaca aucheri*, *Halothamnus bottae*, *Halothamnus iraqensis*, *Salicornia persia*, *Salsola arabica*, and *Salsola villosa*, growing in the Qassim area, the Kingdom of Saudi Arabia. The antidiabetic activity of the hydroalcoholic extracts was assessed using in vitro testing of α-glucosidase and α-amylase inhibitory effects. Among the nine tested extracts, *A. alopecuroides* extract (AAE) displayed potent inhibitory activity against α-glucosidase enzyme with IC_50_ 117.9 µg/mL noting better activity than Acarbose (IC_50_ 191.4 µg/mL). Furthermore, AAE displayed the highest α- amylase inhibitory activity among the nine tested extracts, with IC_50_ 90.9 µg/mL. Based upon in vitro testing results, the antidiabetic activity of the two doses (100 and 200 mg/kg) of AAE was studied in normoglycemic and streptozotocin (STZ)-induced diabetic mice. The effects of the extract on body weight, food and water intakes, random blood glucose level (RBGL), fasting blood glucose level (FBGL), insulin, total cholesterol, and triglycerides levels were investigated. Results indicated that oral administration of the two doses of AAE showed a significant dose-dependent increase (*p* < 0.05) in the body weight and serum insulin level, as well as a significant decrease in food and water intake, RBGL, FBGL, total cholesterol, and triglyceride levels, in STZ-induced diabetic mice, compared with the diabetic control group. Meanwhile, no significant differences of both extract doses were observed in normoglycemic mice when compared with normal control animals. This study revealed a promising antidiabetic activity of the wild plant *A. alopecuroides.*

## 1. Introduction

Plants have long been considered important sources of many medicinally useful drugs, as well as many inspiring chemical moieties. The use of plants as remedies may be traced back at least 6000 years [1]. The research targeting investigation of the biological potential of natural products has proven a great impact on the drug development process [2]. Many successful examples confirm the role of plant-isolated metabolites in the development of drugs, e.g., Atremisnin for treatment of malaria and Vinica alkaloids for treatment of cancer [1,3]. The Kingdom of Saudi Arabia (KSA) is gifted with a wide diversity of flora, including trees, shrubs, and herbs of different plant species that are commonly used for edible or folk medicinal uses [4]. The Qassim region is located in the central part of the Saharo-Arabian floristic region. It was reported to contain around 105 weed species, belonging to 78 genera and 25 families [4,5,6]. Reviewing the literature, we found that most of these species are insufficiently investigated. Accordingly, exploration of the phytochemical contents and biological activities of different plants collected from this area is a promising research idea.

Diabetes mellitus (DM) is a chronic metabolic disorder characterized by elevated blood glucose levels resulting from disturbances in insulin secretion, utilization, or both [7]. Due to its high prevalence and serious complications, it continues to be a major medical concern worldwide [2,8,9]. The development of new antidiabetic medicines from natural sources seems to be an attractive approach due to the limitations of the currently available medications due to safety, efficacy, or cost issues [8]. Plants could manage the elevated blood glucose level through various mechanisms, e.g., blocking the potassium channel of the pancreatic beta cells, providing essential heavy metals for the pancreatic beta cells, inhibition of α-glucosidase and α-amylase, etc. [10].

Postprandial hyperglycemia is one of the most important abnormalities of glucose homeostasis that plays a significant role in the chronic complications associated with type 2 diabetes mellitus (T2DM), e.g., cerebrovascular, cardiovascular, and neuropathy diseases. It has been stated that maintaining a healthy blood glucose level is an important strategy for the treatment of T2DM [11,12]. Accordingly, weight control, a healthy diet, and physical activity are the first lines of treatment. However, if these lifestyle measures fail to maintain normal blood glucose levels, then drug therapy is usually advised [13]. Drugs such as Acarbose act via the inhibition of carbohydrate-hydrolyzing enzymes, e.g., α-amylase and α-glucosidase, and are commonly used for the treatment of T2DM [14]. Pancreatic α-amylase and intestinal α-glucosidase are two important digestive enzymes. Pancreatic α-amylase is a calcium metalloenzyme that catalyzes the hydrolysis of polysaccharides into oligosaccharides, while α-glucosidase is a digestive enzyme that catalyzes the final step in the digestion of carbohydrates acting upon 1, 4-α bonds giving glucose. Accordingly, the suppression of the activity of these enzymes significantly decreases the postprandial blood glucose level in T2DM [11,13].

In light of all the previously mentioned points, the current study was designed to investigate the antidiabetic potential of nine plants collected from Qassim flora by in vitro study of their inhibitory activity against α-amylase and α-glucosidase enzymes as a preliminary step, followed by in vivo testing of the most promising extract.

## 2. Results and Discussion

### 2.1. Percentage Yield of Plant Material Extraction

Aqueous methanol (80%) was used to extract the metabolites content of each plant species, adopting the cold maceration method. Different percentage yields of gummy extracts were harvested at the end of the extraction process. The extraction yields were calculated as follows: *A. alopecuroides* (8.8%), *A. lachnantha* (16.5%), *A. leucoclada* (20.0%), *C. aucheri* (28.0%), *H. bottae* (15.3%), *H. iraqensis* (14.0%), *S. persia* (40.0%), *S. arabica* (19.9%), and *S. villosa* (23.3%). All extracts exhibited better water solubility than in organic solvent, indicating high content of polar constituents.

### 2.2. In Vitro Enzyme Inhibition Assays

Plants have long been used for the treatment of diabetes. Numerous studies investigated the possible mechanisms of action of some plant extracts. Several pathways such as increasing insulin secretion and glucose uptake by various cells and inhibiting glucose production and absorption are suggested as responsible for antidiabetic effects [13]. One of the important approaches approved as a treatment of diabetes is the inhibition of the digestive enzymes, e.g., α-amylase and α-glucosidase, responsible for carbohydrate metabolism, thus decreasing postprandial hyperglycemia. In this regard, the present study investigated the antidiabetic potential of the hydroalcoholic extracts from nine Amaranthaceae plants via assessment of their inhibitory activity against the two enzymes α-amylase and α-glucosidase.

#### 2.2.1. α-Amylase Inhibitory Activity

α-Amylase exists in the saliva and pancreatic juice and is considered as one of the most important enzymes involved in carbohydrates metabolism. Inhibition of the activity of this enzyme results in lowering the elevated postprandial blood glucose level [14]. Testing the inhibitory potential of the nine plant extracts, against the α-amylase enzyme, indicated the highest activity of AAE, with IC_50_ 90.9 µg/mL (Table 1). Additionally, the extracts of *A. lachnantha*, *A. leucoclada*, and *S. villosa* expressed a good level of activity, with IC_50_ 181.8, 214.2, and 374.2 µg/mL, respectively. A mild level of activity was observed for the two *Halothamnus* species: *H. bottae* and *H. iraqensis*. Other extracts displayed very weak activity (Table 1). Gjeridane et al. (2015) [15] investigated the α-amylase inhibitory activity of six Algerian plants and concluded that *A. alopecuroides* extract exhibited a mixed non-competitive enzyme inhibition. Herein, the current study confirmed the significant activity of *A. alopecuroides* and estimated its IC_50_ value that acknowledged the best activity of AAE among the nine tested extracts. Interestingly, none of the other eight tested species was previously reported for α-amylase inhibitory activity. However, the enzyme inhibitory activities of other species, e.g., some *Anabasis* species such as *A. aretioides* and *A. articulate* [15,16]; *Atriplex* species, e.g., *A. lasiantha* [17]; *Cornulaca* species, e.g., *C.monacantha* [18], as well as *Salsola* species such as *S. kali, S. soda*, *S. oppositifolia* [19], *S. imbricate*, and *S. cyclophylla* [20], were previously reported.

#### 2.2.2. α-Glucosidase Inhibitory Activity

The α-Glucosidase enzyme is also one of the most important digestive enzymes, located in the small intestine. Its role in the digestion of complex carbohydrates into simple absorbable sugars makes it an important target for antidiabetic drugs. Drugs with inhibitory potential against this enzyme could decrease the elevated postprandial glucose levels, hence preventing the progression of diabetes [13,21]. Results (Table 1) revealed the potent inhibitory activity of AAE against the α-glucosidase enzyme, which exceeded that for the standard, Acarbose, as indicated by its IC_50_ value 117.9 µg/mL, compared with 191.4 µg/mL of Acarbose. Interestingly were also the α-glucosidase inhibitory activity of the two species—*H. bottae* and *A. lachnantha*—expressed as IC_50_ values 192.8 and 201.2 µg/mL, respectively. The other tested species—namely, *S. villosa, A. leucoclada, S. arabica, S. persia, H. iraqensis,* and *C. aucheri*, exhibited mild-to-very-weak inhibitory activity against this enzyme (Table 1). These results represented the first report for the antienzyme activity of the abovementioned nine Amaranthaceae plants. However, a single previous study investigated the kinetic behavior of the enzyme inhibitory activity of six plants including *A. alopecuroides* phenolic fraction [15]. These results greatly supported our findings concerning the potent activity of AAE against the α-glucosidase enzyme.

Based upon the aforementioned in vitro results, AAE was indicated for possible antidiabetic activity and selected for further confirmation of its antidiabetic potential adopting an in vivo streptozotocin (STZ)-induced diabetic model.

### 2.3. Acute Oral Toxicity Study

The acute toxicity study of AAE did not show mortality in the animals at the limit dose of 2000 mg/kg during the observation period. The animals did not show any signs of irritation, respiratory distress, diarrhea, convulsions, or restlessness. Thus, AAE is considered safe for mice at the studied doses.

### 2.4. In Vivo Antidiabetic Testing

Streptozotocin (STZ) is a diabetogenic agent commonly used for inducing experimental diabetes models due to its effectiveness and high reproducibility. It acts via the DNA alkylating activity of its methyl-nitroso urea moiety, followed by the release of nitric oxide from the nitroso group and generation of reactive oxygen species, resulting in pancreatic β-cell death [7,22]. The present study employed STZ at a dose of 55 mg/kg body weight, for 5 consecutive days, to induce diabetes [23]. Glibenclamide (GLC) is a second-generation sulfonylurea drug commonly used in the treatment of non-insulin-dependent diabetes. It exerts its antidiabetic effect via stimulating insulin secretion, inhibiting insulin degradation in the vascular endothelial cells of the liver, as well as insulin-independent blood-glucose-lowering activities. Herein, GLC (5 mg/kg) was used as a positive antidiabetic drug [24,25]. The effect of AAE on physical and biochemical parameters was measured in normoglycemic and diabetic mice. Based upon the acute toxicity results, two doses of AAE, 100 and 200 mg/kg, were chosen for testing the antidiabetic activity of the plant. It is worth mentioning that this is the first in vivo study of *A. alopecuroides*.

#### 2.4.1. Effect of AAE on Body Weight and Water and Food Intakes in Normoglycemic and STZ-Induced Diabetic Mice

Diabetes mellitus induced by STZ is characterized by body weight loss, which could be attributable to the inability of cells to use glucose, lipolysis occurring in adipose tissue, and protein breakdown, resulting in skeletal muscle wasting [8,10]. Additionally, STZ increases both food intake and water intake. Herein, diabetic control animals displayed a significant reduction in body weight (−16.6%) when compared with normal control animals. Additionally, significant increases in food and water intakes were observed in STZ-treated animals (14.5 ± 1.1 and 21.7 ± 1.6, respectively) in comparison with normal animals (5.3 ± 0.6 and 4.3 ± 0.5, respectively), indicating successful induction of diabetes (Table 2). Treatments with AAE, in low and high doses, significantly ameliorated body weight loss in diabetic mice (38.6% and 44.1%, respectively), as well as food intake (8.1 ± 0.9 and 4.1 ± 0.3, respectively) and water intake (11.4 ± 0.4 and 6.9 ± 1.4, respectively), in a dose-dependent manner (Table 2). On the other hand, both doses of AAE did not produce any significant changes in body weight or food intake in normoglycemic animals, as compared with the normal control group. Glibenclamide-treated group (positive control) displayed significant improvements in body weight (40.8%), food intake (6.12 ± 1.9), and water intake (7.28 ± 2.4), compared with the STZ-treated group. Collectively, these results indicated a dose-dependent, protective effect of AAE comparable to that of glibenclamide. This effect could be attributed to its ability to reduce hyperglycemia in diabetic mice [26].

#### 2.4.2. Effect of AAE on RBGL and FBGL in Normoglycemic and STZ-Induced Diabetic Mice

**Figure 1:**
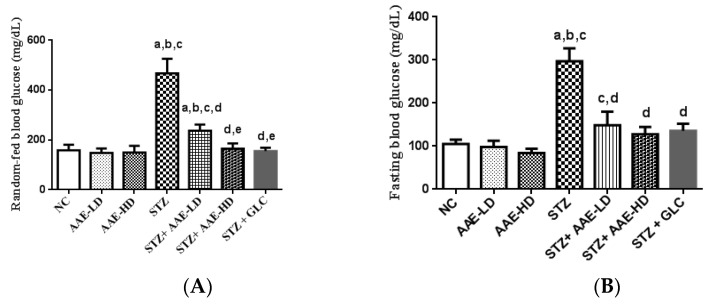
Effect of hydroalcoholic AAE on RBGL (**A**) and FBGL (**B**) with or without STZ in mice. Data in the figure represent mean ± SEM. Data were analyzed using one-way ANOVA, followed by Tukey–Kramer post hoc test. ^a^ Significantly different from NC group; ^b^ significantly different from AAE-LD; ^c^ significantly different from AAE-HD; ^d^ significantly different from STZ-treated group; ^e^ significantly different from STZ + AAE-LD at *p* < 0.05. ANOVA: analysis of variance; NC: normal control; AAE-LD: *A. alopecuroides*—low dose; AAE-HD: *A. alopecuroides*—high dose; STZ: streptozotocin; GLC: glibenclamide; SEM: standard error of the mean.

The increase in the blood glucose level is an important feature recorded in diabetic animals [9]. STZ-injected mice showed significant elevation in RBGL (467 ± 39.4 mg/dL) and FBGL (297 ± 15.5 mg/dL), compared with control (158 ± 16.0 and 105 ± 5.0 mg/dL, respectively), thus confirming the induction of diabetes in mice (Figure 1A,B, respectively). Ingestion of AAE to normoglycemic mice caused differences in RBGL and FBGL (148 ± 17.5 and 98 ± 7.3 mg/dL, respectively) in low dose; and (149 ± 16.5 and 84 ± 5.0 mg/dL, respectively) in high dose. The observed decrease in BGLs of normoglycemic mice was found to be non-significant from RBGL and FBGL in normal control mice. These findings stated that AAE, in low and high doses, did not produce hypoglycemia in normal mice. Diabetic animals treated with AAE exhibited, a significant, dose-dependent amelioration of the elevated RBGL and FBGL in low dose (237 ± 16.3 and 149 ± 15.5 mg/dL, respectively) and high dose (165 ± 11.6 and 128 ± 8.3 mg/dL, respectively), when compared with control diabetic mice. These results were found to be non-significant from the results of the glibenclamide-treated mice group (Figure 1A,B). Accordingly, it could be stated that AAE exhibited antihyperglycemic activity in diabetic mice with no hypoglycemic effect on normoglycemic mice. The antihyperglycemic effect, with no risk of hypoglycemia, was similarly reported for many plant extracts, e.g., *Nymphaea stellata* [27], *Rubus Erlangeri* [28], and *Justicia Schimperiana* [29].

#### 2.4.3. The Effect of AAE on Serum Insulin in Normoglycemic and STZ-Induced Diabetic Mice

**Figure 2:**
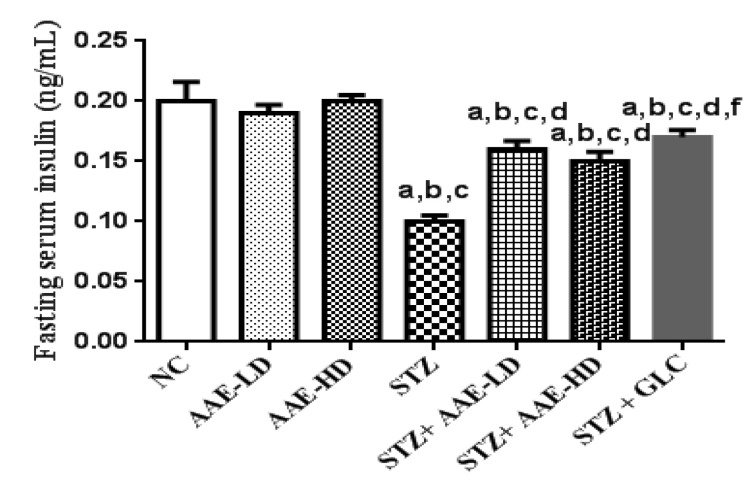
Effect of hydroalcoholic AAE on serum insulin in the presence or absence of STZ in mice. Data in the figure represent mean ± SEM. Data were analyzed using one-way ANOVA, followed by Tukey–Kramer post hoc test. ^a^ Significantly different from NC group; ^b^ significantly different from AAE-LD; ^c^ significantly different from AAE-HD; ^d^ significantly different from STZ-treated group; ^f^ significantly different from STZ + AAE-HD at *p* < 0.05. ANOVA: analysis of variance; NC: normal control; AAE-LD: *A. alopecuroides*—low dose; AAE-HD: *A. alopecuroides*—high dose; STZ: streptozotocin; GLC: glibenclamide; SEM: standard error of the mean.

STZ is a diabetogenic drug commonly used in experimental models for the induction of diabetes. Its action is mediated through the destruction of β cells in islets of Langerhans of the pancreas, accordingly, a significant decrease in serum insulin level is predicted with subsequent hyperglycemia [30]. In the present study, AAE produced a significant improvement in the elevated RBGL and FBGL; therefore, the effect of AAE on serum insulin level was further assessed. Results indicated that STZ caused a significant reduction in serum insulin level, thus confirming the induction of diabetes. Oral administration of AAE, in both low and high doses, produced a significant increase in serum insulin level in STZ-induced diabetic mice, noting that the extract did not display any changes in serum insulin level of normoglycemic mice (Figure 2). This activity might be due to the protective effect of AAE on pancreatic β cells against the damage induced by STZ. This conclusion is supported by a previous study that considered the stimulation of insulin secretion as one of the proposed mechanisms of antidiabetic activity of plant extracts [30].

#### 2.4.4. The Effect of AAE on Plasma Cholesterol and Triglycerides in Normoglycemic and STZ-Induced Diabetic Mice

**Figure 3:**
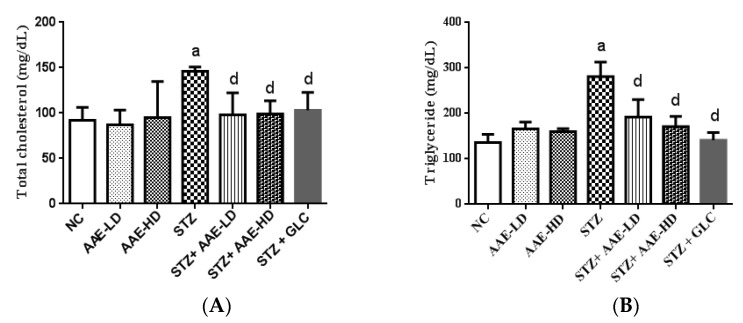
Effect of hydroalcoholic AAE on total cholesterol (**A**) and triglycerides (**B**) in the presence or absence of STZ in mice. Data in the figure represent mean ± SEM. Data were analyzed using one-way ANOVA, followed by Tukey–Kramer post hoc test. ^a^ Significantly different from NC group; ^d^ significantly different from STZ-treated group at *p* < 0.05. ANOVA: analysis of variance; NC: normal control; AAE-LD: *A. alopecuroides*—low dose; AAE-HD: *A. alopecuroides*—high dose; STZ: streptozotocin; GLC: glibenclamide; SEM: standard error of the mean.

Dyslipidemia is one of the common complications of DM, where there are increased levels of cholesterols and triglycerides [9,31]. This increase could be due to disturbances in hormone-sensitive enzymes, e.g., lipase [31]. Herein, STZ-induced diabetic mice displayed a significant increase in plasma cholesterol (146 ± 2.9 mg/dL), compared with the control group (92 ± 7.0 mg/dL) (Figure 3A). Administration of AAE to STZ-induced diabetic mice in low and high doses restored the level of cholesterol to almost its normal value (98 ± 12.0 and 99 ± 7.2 mg/dL, respectively) signifying the hypolipidemic effect of AAE on diabetic animals. However, ingestion of AAE to normal mice in low and high doses did not show any significant changes in plasma cholesterol level (87 ± 8.0 and 95 ± 20.0 mg/dL, respectively), compared with control mice (Figure 3A). Similarly, a significant increase in plasma triglycerides was found in STZ-treated mice (281 ± 15.9 mg/dL), compared with the control group (136 ± 9.0 mg/dL) (Figure 3B). This increase was significantly improved by the administration of AAE in low (192 ± 19.2 mg/dL) and high doses (171 ± 11.5 mg/dL) to diabetic mice. On the other hand, normal mice treated with AAE did not display significant changes in mice at low (166 ± 7.5 mg/dL) or high doses (160 ± 3.4 mg/dL), when compared with control mice (Figure 3B). The results of AAE (low and high) doses were non-significantly different from those recorded for the positive control drug, glibenclamide (Figure 3A,B). Reviewing the relevant literature, we found several reports stating the hypolipidemic effect as one of the possible mechanisms for the antidiabetic activity of some plants [31]. Accordingly, and based upon the current results, the hypolipidemic effect of AAE might contribute to its antidiabetic potential.

Therefore, all the above mentioned in vitro and in vivo results suggested that the antidiabetic effect of AAE might be through multitarget mechanisms. In this context, a number of medicinal plants were reported to exhibit antidiabetic activity through variable mechanisms—namely, *Prosopis cineraria*, *Thymus schimperi*, *Rumex rothschildianus*, *Punica granatum*, and *Calpurnia aurea* [8,9,10,31,32].

## 3. Materials and Methods

### 3.1. Drugs, Chemicals, and Instruments

α-Amylase, α-glucosidase, acarbose, phosphate-buffered solution, p-nitrophenyl-α-d-glucopyranoside (PNPG), streptozotocin, glibenclamide, and methanol were obtained from Sigma Chemical (St. Louis, MO, USA). Plasma total cholesterol and triglycerides assay kits were purchased from Nanjing Jiancheng (Nanjing, China). Mouse insulin ELISA kit was obtained from Biovision (Milpitas, CA, USA).

### 3.2. Plant Material Collection and Preparation

Nine plants representing six genera of family Amaranthaceae—Agathophora alopecuroides var. papillosa, *Anabasis lachnantha*, *Atriplex leucoclada*, *Cornulaca aucheri*, *Halothamnus bottae*, *Halothamnus iraqensis*, *Salicornia persia ssp. Iranica*, *Salsola arabica,* and *Salsola villosa*—were collected from the Qassim region in the northcentral Saudi Arabia during October–December 2020. The taxonomic identities of the plants were confirmed by Ibrahim Aldakhil, botanical expert, Qassim Area, and voucher samples were kept at the College of Pharmacy, Qassim University (Buraydah, Qassim, Saudi Arabia).

### 3.3. Preparation of Plant Material Extract

The aerial parts of the nine plants were separately washed with distilled water and then dried in the shade for two weeks. The dried plants were individually powdered to a fine powder. A total of 300 grams of each of the powdered plant materials was separately macerated in 80% methanol (3 times × 500 mL, each time plants were macerated for 24 h) with frequent shaking. Each extract was separately filtered and dried at 40 °C using a rotatory evaporator (R-215, Buchi, Flawil, Switzerland). The dried extracts were then kept in amber-colored vials at 4 °C till used in the experiments.

### 3.4. In Vitro Enzyme Inhibition Assays

#### 3.4.1. α-Glucosidase Inhibitory Activity

α-Glucosidase inhibitory activity was determined spectrophotometrically according to the method described by Feng et al. 2011 [21]. Briefly, 20 µL of α-glucosidase enzyme solution (0.8 U/mL in 0.01 M potassium phosphate buffer) was mixed with 120 µL of the tested aqueous extract, and preincubated, for 15 min, at 37 °C prior to initiation of the reaction by adding the substrate. Afterward, p-nitrophenyl-β-glucopyranoside (PNPG) solution (20 µL) was added and then incubated together at 37 °C, for 15 min. After incubation time, 80 µL Na_2_CO_3_ (0.2 M) was added to the test tube, to stop the reaction, and the amount of PNP released was quantified at 405 nm.

#### 3.4.2. α-Amylase Inhibitory Activity

α-Amylase inhibitory activity was carried out according to the iodine–starch method described by Kotowaroo et al. (2006) [33]. In a 96-well plate, reaction mixture containing 50 µL alpha-amylase (0.5 mg/mL) in phosphate buffer (50 mM, Ph = 6.8) was mixed with 50 µL the plant extracts. The mixtures were preincubated at 37 °C for 10 min. After preincubation, 100 µL soluble starch (1%) was added as a substrate and incubated further at 37 °C for 20 min. The reaction was stopped by adding 20 µL 1NHCl, followed by the addition of a 50 µL iodine reagent. The intensity of the blue color was measured at 620 nm using Thermo Scientific Multiskan^®^ EX (Thermo scientific, Waltham, MA, USA). Acarbose at various concentrations (3–3000 µg/mL) was included as a reference control. Percent inhibition was calculated as follows:

Inhibition (%) = (A_Negative control_ − A_Test_/A_Negative control_) × 100, where A is absorbance. The results were calculated and expressed as the concentration of extract resulting in 50% inhibition of enzyme activity (IC_50_) values. The (IC_50_) values were determined from a plot of percentage inhibition against log extract concentrations using GraphPad Prism version 6.0 (GraphPad Software, San Diego, CA, USA) (Figures S1 and S2).

#### 3.4.3. Experimental Animals

BALB/c male albino mice weighing 22–27 g were obtained from Nahda University Animal Facility. Mice were allowed standard fed (El-Nasr Company, Abou-Zaabal, Cairo, Egypt) and water access ad libitum. Mice were acclimatized for one week at 22 ± 2 °C, 55 ± 10% humidity with a 12 h light/dark cycle.

### 3.5. Acute Toxicity Study

Acute oral toxicity of the AAE was evaluated using the “up-and-down” test method in mice at a single dose of 2000 mg/kg based on the limit test recommendations of Organization for Economic Development (OECD) No. 425 Guideline [34]. Six male mice were used in each dose in our study. On day one of the experiment, AAE extract in a dose of 2000 mg/kg was given orally to one Swiss albino mouse fasted for 3 h. The mouse was then observed individually at least one time during the first 30 min and regularly for the upcoming 24 h, paying more attention during the first 4 h. No mortality was observed in the first mouse; hence, the other five fasting mice were sequentially given a single dose (2000 mg/kg) of AAE and then observed in the same manner for 14 days, for any sign of toxicity and mortality. Careful observations were carried out for various manifestations of salivation, diarrhea, tremor, lethargy, convulsions, respiratory distress, and sleeping.

### 3.6. Antidiabetic Activity Model

The study design was ethically approved by Nahda University Ethical Committee, Beni-suef, Egypt (NUB-083-2021). Experimental diabetes was induced by intraperitoneal injection of STZ at a dose of 55 mg/kg, dissolved in sodium citrate buffer (50 mmol/L, pH 4.5), once daily for 5 consecutive days [23]. Animals with random-fed blood glucose level (RBGL) > 400 mg/dL and fasting blood glucose level (FBGL) > 200 mg/dL were considered diabetic. Glibenclamide (5 mg/kg) was used as a standard drug. RBGL, FBGL, and body weight were used to assess the antidiabetic activity. Mice were classified into the following seven groups (*n* = 10):

▪ The normal control (NC) group received sodium citrate buffer (0.2 mL/mouse, i.p.);
▪ The second group received AAE (100 mg/kg p.o.) from the first day till day 15;
▪ The third group received AAE (200 mg/kg p.o.) from the first day till day 15;
▪ The fourth group received STZ 55 mg/kg, i.p., once daily for 5 consecutive days, starting from the first day;
▪ The fifth group (STZ + AAE-LD) received AAE (100 mg/kg p.o.) for 15 days, beginning from the start of the experiment, with STZ for 5 consecutive days;
▪ The sixth group (STZ + AAE-HD) received AAE (200 mg/kg p.o.) for 15 days, beginning from the start of the experiment, with STZ for 5 consecutive days;
▪ The seventh group (STZ + GLC) received GLC (5mg/kg p.o.) for 15 days, beginning from the start of the experiment, with STZ for 5 consecutive days.

### 3.7. Determination of Body Weight Change and Blood Glucose

Calculation of the changes in the body weight was assessed by the following equation: % Change body weight = weight of animals at day 15 (final body weight) − weight of animals at day 0 (initial body weight)/initial body weight × 100. To confirm the induction of diabetes, blood samples from different groups were tested for blood glucose after 15 days of starting the experiment. Direct measurement of blood glucose level was performed with one drop of blood using a glucometer (Uright, Taiwan).

### 3.8. Determination of Plasma Total Cholesterol and Triglycerides

After 15 days of starting the experiment, blood samples were collected from the retro-orbital sinus from all animals after a 12 h fasting time. Plasma samples were isolated after collecting blood samples in citrate buffered tubes, centrifuged at 4000 rpm for 15 min. The total cholesterol and triglyceride levels in plasma were evaluated using colorimetric assays and according to the manufacturer’s instructions (Jianchen, Nanjing, China).

### 3.9. Determination of Serum Insulin

Blood samples were collected in centrifuge tubes then centrifuged for 20 min at 4000 rpm for serum collection. To confirm the induction of diabetes, serum samples from all groups were tested for serum insulin after 15 days of starting the experiment. Serum insulin was assessed using mouse insulin ELISA kit Biovision Inc. (Milpitas, CA, USA), following the manufacturer’s instructions.

### 3.10. Statistical Analysis

Data in the current study were represented as mean ± SEM and statistically using GraphPad Prism 6 software (GraphPad Software, San Diego, CA, USA). In order to compare all groups, a one-way analysis of variance (ANOVA), followed by Tukey–Kramer post-ANOVA test, was used, and *p* < 0.05 was considered statistically significant.

## 4. Conclusions

In conclusion, the present study assessed for the first time the inhibitory activity of hydroalcoholic extracts of the nine Amaranthaceae wild plants against two carbohydrate-metabolizing enzymes and indicated *Agathophora alopecuroides* extract (AAE) as a potent α-glucosidase inhibitor better than Acarbose. Furthermore, in vivo testing of AAE confirmed its antidiabetic efficacy through multitarget mechanisms, including antihyperglycemic, increased insulin production, and hypolipidemic effects. The observed antidiabetic activity of AAE might be due to the presence of bioactive phytoconstituents in AAE. Accordingly, more studies are required to isolate and characterize the bioactive phytoconstituents responsible for this activity.

## Figures and Tables

**Table 1:** IC_50_ Values of the crude extracts from the nine plants against α-amylase and α-glucosidase.

Ser.	Plant Species	IC_50_ α-Amylase (µg/mL)	IC_50_ α-Glucosidase (µg/mL)
1	*Agathophora alopecuroides* (AAE)	90.9 ± 1.15	117.9 ± 1.09
2	*Anabasis lachnantha*	181.8 ± 1.18	201.2 ± 1.10
3	*Atriplex leucoclada*	214.2 ± 1.21	427.7 ± 1.04
4	*Cornulaca aucheri*	1984.0 ± 1.11	1194.5 ± 1.08
5	*Halothamnus bottae*	502.9 ± 1.12	192.8 ± 1.05
6	*Halothamnus iraqensis*	929.0 ± 1.11	1019.0 ± 1.07
7	*Salicornia persia*	3653 ± 1.11	638.6 ± 1.11
8	*Salsola arabica*	1509 ± 1.16	531.2 ± 1.08
9	*Salsola villosa*	374.2 ± 1.05	386.7 ± 1.06
Standard	Acarbose	53.3 ± 1.12	191.4 ± 1.14

IC_50_ values are expressed as mean ± SEM, SEM: standard error of the mean.

**Table 2:** Effect of AAE extract on biochemical and physical characteristics of study groups.

	NC	AAE (100 mg/kg)	AAE (200 mg/kg)	STZ	STZ + AAE-LD	STZ + AAE-HD	STZ + GLC
Initial BW * (g)	25.9 ± 0.5	25.0 ± 1.8	26.1 ± 2.8	26.7 ± 1.4	25.1 ± 1.9	24.7 ± 0.7	25.0 ± 2.1
Final BW * (g)	37.5 ± 1.4	34.0 ± 3.0	36.0 ± 1.5	22.3 ± 1.4 ^abc^	34.8 ± 2.4 ^d^	35.6 ± 2.9 ^d^	35.2 ± 1.8 ^d^
% Change	44.8%	36.0%	37.9%	−16.5%	38.6%	44.1%	40.8%
Food intake # (g/mouse/day)	5.3 ± 0.6	4.6 ± 0.2	6.5 ± 0.4	14.5 ± 1.1 ^abc^	8.1 ± 0.9 ^d^	4.1 ± 0.3 ^d^	6.1 ± 1.9 ^d^
Water intake # (ml/mouse/day)	4.3 ± 0.5	5.5 ± 0.8 ^a^	4.4 ± 0.7	21.7 ± 1.6 ^abc^	11.4 ± 0.4 ^bcd^	6.9 ± 1.4 ^d^	7.3 ± 2.4 ^d^

Data are expressed as mean ± SEM. Multiple comparisons were performed using a one-way ANOVA test, followed by Tukey–Kramer post hoc test. ^a^ Significantly different from NC group; ^b^ significantly different from AAE-LD; ^c^ significantly different from AAE-HD; ^d^ significantly different from STZ-treated group (*p* < 0.05). * Number of animals= 8–10; # Number of replicates= 3. ANOVA: analysis of variance; BW: body weight; NC: normal control; AAE-LD: *A. alopecuroides*—low dose; AAE-HD: *A. alopecuroides*—high dose; GLC: glibenclamide; SEM: standard error of the mean.

## Data Availability

Data are contained within the article.

## Supplementary Materials

The following supporting information can be downloaded, Figure S1: Results of α-Amylase inhibitory activity of the nine tested extracts. Figure S2: Results of α-Glucosidase inhibitory activity of the nine tested extracts.

## Sample Availability

Samples of the tested extracts are available from the authors.

## References

[1] Haidan Y., Qianqian M., Ye L., Guangchun P. (2016). The Traditional Medicine and Modern Medicine from Natural Products. Molecules.

[2] El Moussaoui A., Mechchate H., Bourhia M., Es-Safi I., Salamatullah A.M., Alkaltham M.S. (2021). Glycemic control potential of chemically characterized extract from withania frutescens l. Roots in severe diabetes-induced mice. Appl. Sci..

[3] Cragg G.M., Newman D.J. (2013). Natural products: A continuing source of novel drug leads. Biochim. Biophys. Acta.

[4] Aati H., El-Gamal A., Shaheen H., Kayser O. (2019). Traditional use of ethnomedicinal native plants in the Kingdom of Saudi Arabia. J. Ethnobiol. Ethnomed..

[5] El-Ghazali G.E., Al-Khalifa K.S., Saleem G.A., Abdallah E.M. (2010). Traditional medicinal plants indigenous to Al-Rass province, Saudi Arabia. J. Med. Plants Res..

[6] Ghazali G., Al-Soqeer A. (2013). Checklist of the Weed Flora of Qassim Region, Saudi Arabia. Aust. J. Basic Appl. Sci..

[7] Alema N.M., Periasamy G., Sibhat G.G., Tekulu G.H., Hiben M.G. (2020). Antidiabetic activity of extracts of terminalia brownii fresen. Stem bark in mice. J. Exp. Pharmacol..

[8] Belayneh Y.M., Birru E.M. (2018). Antidiabetic activities of hydromethanolic leaf extract of calpurnia aurea (Ait.) benth. Subspecies aurea (Fabaceae) in mice. Evid.-Based Complement. Altern. Med..

[9] Pottathil S., Nain P., Morsy M.A., Kaur J., Al-Dhubiab B.E., Jaiswal S. (2020). Mechanisms of antidiabetic activity of methanolic extract of punica granatum leaves in nicotinamide/streptozotocin-induced type 2 diabetes in rats. Plants.

[10] Taye G.M., Bule M., Gadisa D.A., Teka F., Abula T. (2020). In Vivo antidiabetic activity evaluation of aqueous and 80% methanolic extracts of leaves of thymus schimperi (Lamiaceae) in alloxan-induced diabetic mice. Diabetes Metab. Syndr. Obes. Targets Ther..

[11] Mumtaz M.W., Al-Zuaidy M.H., Hamid A.A., Danish M., Akhtar M.T., Mukhtar H. (2018). Metabolite profiling and inhibitory properties of leaf extracts of Ficus benjamina towards α-glucosidase and α-amylase. Int. J. Food Prop..

[12] Alqahtani A.S., Hidayathulla S., Rehman T., Elgamal A.A., Dib R.A., El Alajmi M.F. (2020). Alpha-amylase and alpha-glucosidase enzyme inhibition and antioxidant potential of 3-oxolupenal and katononic acid isolated from Nuxia oppositifolia. Biomolecules.

[13] El-Abhar H.S., Schaalan M.F. (2014). Phytotherapy in diabetes: Review on potential mechanistic perspectives. World J. Diabetes.

[14] Saha S., Verma R. (2012). Inhibitory potential of traditional herbs on α-amylase activity. Pharm. Biol..

[15] Djeridane A., Hamdi A., Bensania W., Cheifa K., Lakhdari I., Yousfi M. (2015). The in vitro evaluation of antioxidative activity, α-glucosidase and α-amylase enzyme inhibitory of natural phenolic extracts. Diabetes Metab. Syndr. Clin. Res. Rev..

[16] Berrani A., Marmouzi I., Kharbach M., Bouyahya A., El Hamdani M., El Jemli M. (2019). Anabasis aretioides Coss. & Moq. phenolic compounds exhibit in vitro hypoglycemic, antioxidant and antipathogenic properties. J. Basic Clin. Physiol. Pharmacol..

[17] Zohra T., Ovais M., Khalil A.T., Qasim M., Ayaz M., Shinwari Z.K. (2019). Bio-guided profiling and HPLC-DAD finger printing of Atriplex lasiantha Boiss. BMC Complement. Altern. Med..

[18] El-Manawaty M.A., Gohar L. (2018). In vitro alpha-glucosidase inhibitory activity of egyptian plant extracts as an indication for their antidiabetic activity. Asian J. Pharm. Clin. Res..

[19] Tundis R., Loizzo M.R., Statti G.A., Menichini F. (2007). Inhibitory effects on the digestive enzyme α-amylase of three Salsola species (Chenopodiaceae) in vitro. Pharmazie.

[20] Al-Omar M.S., Mohammed H.A., Mohammed S.A.A., Abd-Elmoniem E., Kandil Y.I., Eldeeb H.M. (2020). Anti-microbial, anti-oxidant, and α-amylase inhibitory activity of traditionally-used medicinal herbs: A comparative analyses of pharmacology, and phytoconstituents of regional halophytic plants’ diaspora. Molecules.

[21] Feng J., Yang X.W., Wang R.F. (2011). Bio-assay guided isolation and identification of α-glucosidase inhibitors from the leaves of Aquilaria sinensis. Phytochemistry.

[22] Goud J.B., Dwarakanath V., Chikkaswamy B.K. (2015). Streptozotocin A Diabetogenic Agent in Animal Models. Int. J. Pharm. Pharm. Res..

[23] Kim Y.H., Kim Y.S., Kang S.S., Cho G.J., Choi W.S. (2010). Resveratrol inhibits neuronal apoptosis and elevated Ca^2+^/ calmodulin-dependent protein kinase II activity in diabetic mouse retina. Diabetes.

[24] Sokolovska J., Isajevs S., Sugoka O., Sharipova J., Paramonova N., Isajeva D., Rostoka E., Sjakste T., Kalvinsh I., Sjakste N. (2012). Comparison of the Effects of Glibenclamide on Metabolic Parameters, GLUT1 Expression, and Liver Injury in Rats with Severe and Mild Streptozotocin-Induced Diabetes Mellitus. Medicina.

[25] Sai Laxmi M., Venkatesham A., Rama Narsimha Reddy A., Shankaraiah P., Krishna D.R., Narsihma Reddy Y. (2009). Glibenclamide Therapy in Type 2 Diabetes. Int. J. Pharm. Sci. Nanotechnol..

[26] Ahmad W., Khan I., Khan M.A., Ahmad M., Subhan F., Karim N. (2014). Evaluation of antidiabetic and antihyperlipidemic activity of Artemisia indica linn (aeriel parts) in Streptozotocin induced diabetic rats. J. Ethnopharmacol..

[27] Rajagopal K., Sasikala K., Ragavan B. (2008). Hypoglycemic and antihyperglycemic activity of Nymphaea stellata flowers in normal and alloxan diabetic rats. Pharm. Biol..

[28] Ayele A.G., Kumar P., Engidawork E. (2021). Antihyperglycemic and hypoglycemic activities of the aqueous leaf extract of Rubus Erlangeri Engl (Rosacea) in mice. Metab. Open.

[29] Tesfaye A., Makonnen E., Gedamu S. (2016). Hypoglycemic and antihyperglycemic activity of aqueous extract of Justicia Schimperiana leaves in normal and streptozotocin-induced diabetic mice. Int. J. Pharma. Sci. Res..

[30] Salahuddin M., Jalalpure S.S., Gadge N.B. (2010). Antidiabetic activity of aqueous bark extract of Cassia glauca in streptozotocin-induced diabetic rats. Can. J. Physiol. Pharmacol..

[31] Soni L.K., Dobhal M.P., Arya D., Bhagour K., Parasher P., Gupta R.S. (2018). In vitro and in vivo antidiabetic activity of isoaltrd fraction of Prosopis cineraria against streptozotocin-induced experimental diabetes: Amechanistic study. Biomed. Pharmacother..

[32] Jaradat N., Hawash M., Dass G. (2021). Phytochemical analysis, in-vitro antiproliferative, anti-oxidant, anti-diabetic, and anti-obesity activities of Rumex rothschildianus Aarons. extracts. BMC Complement. Med. Ther..

[33] Kotowaroo M.I., Mahomoodally M.F., Gurib-Fakim A., Subratty A.H. (2006). Screening of traditional antidiabetic medicinal plants of Mauritius for possible α-amylase inhibitory effects in vitro. Phyther. Res..

[34] OECD (2008). Test No. 425: Acute Oral Toxicity: Up-and-down Procedure, OECD Guidelines for the Testing of Chemicals. https://ntp.niehs.nih.gov/iccvam/suppdocs/feddocs/oecd/oecdtg425.pdf.

