# Supplementary material for: The Glycemic Control Potential of Some Amaranthaceae Plants, with Particular Reference to In Vivo Antidiabetic Potential of Agathophora alopecuroides
Source: Molecules. 2022 Feb 1;27(3):973. doi: 10.3390/molecules27030973 (PMC8839903; doi:10.3390/molecules27030973)
Supplement: Supplementary file 1 [file molecules-27-00973-s001.zip › molecules-1549441-supplementary.pdf]

Supplementary Data

Glycemic Control Potential of Some Amaranthaceae Plants; With Particular Reference to *In Vivo* Antidiabetic Potential of *Agathophora alopecuroides*

Elham Amin [1,2,*], Mohamed Abdelbakky [3,4], Mostafa Assem Darwish [5], Hamdoon Mohamed [1,6], Sridevi Chigrupati [1], Kamal Ahmad Qureshi [7], Marwa Hassan [2]

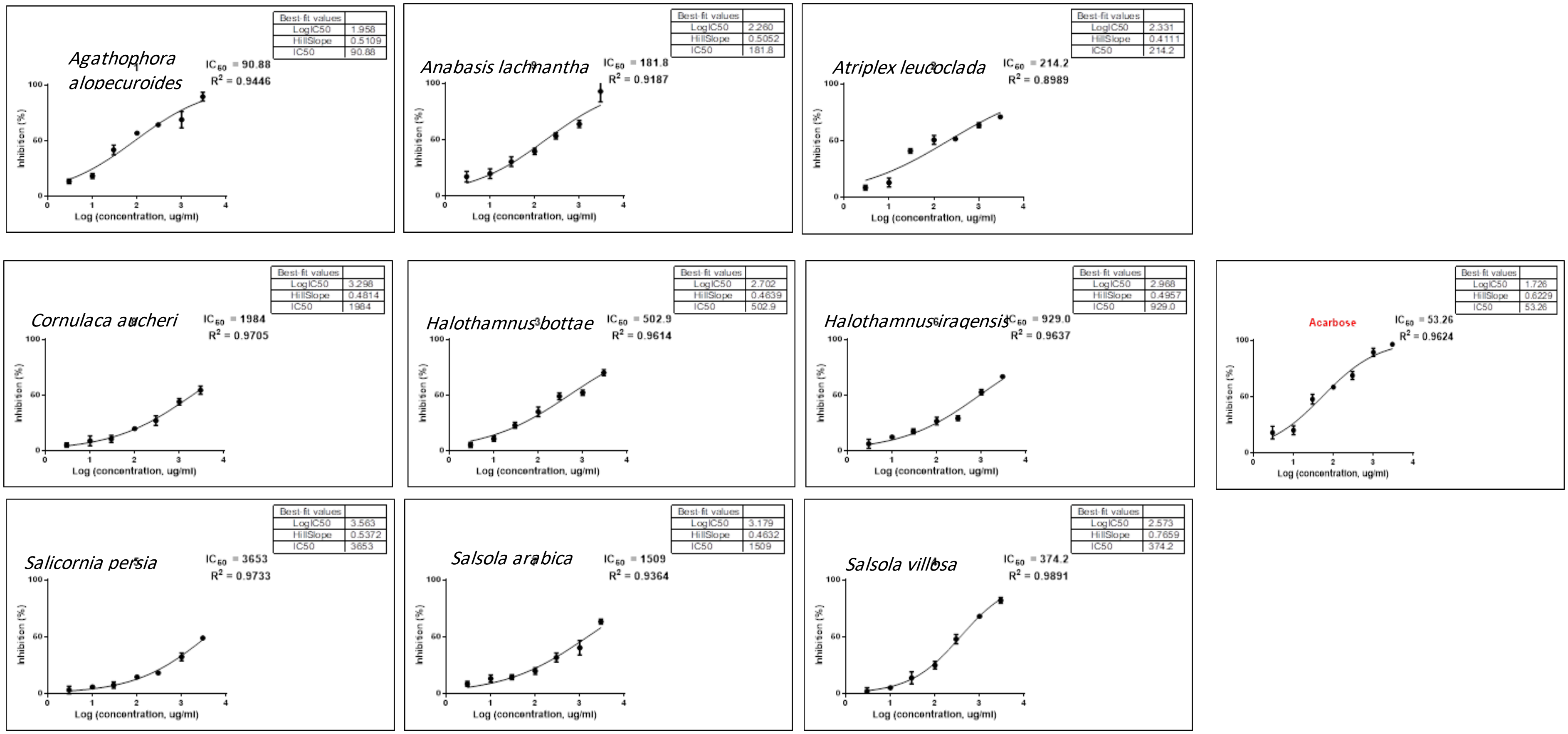

Figure S1: Results of α-Amylase inhibitory activity of the nine tested extracts.

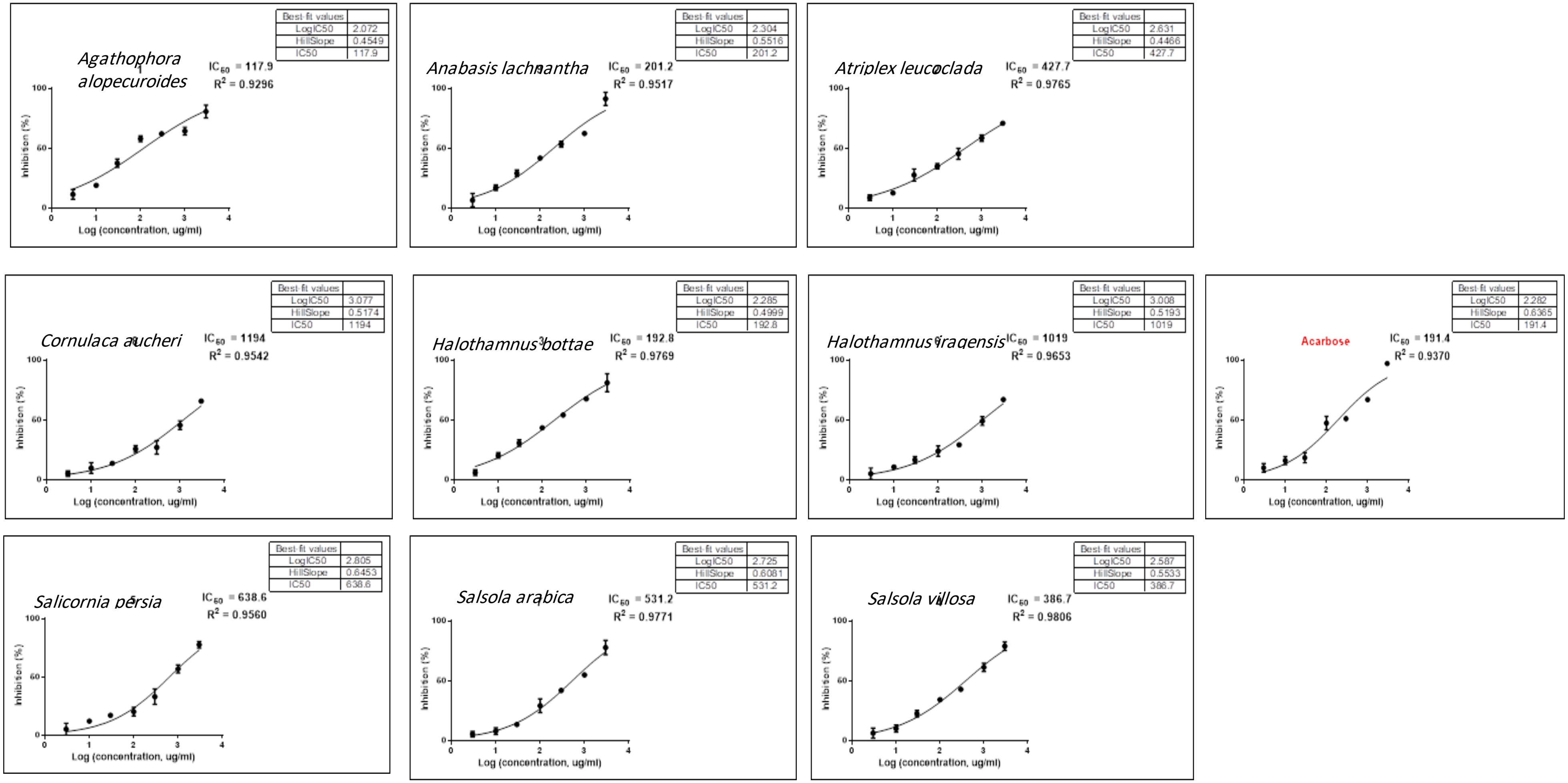

Figure S2: Results of α-Glucosidase inhibitory activity of the nine tested extracts.
